# RT-PCR Testing of Organ Culture Medium for Corneal Storage Fails to Detect SARS-CoV-2 Infection Due to Lack of Viral Replication

**DOI:** 10.3390/pathogens11020133

**Published:** 2022-01-22

**Authors:** Lisa Müller, Philipp Niklas Ostermann, Heiner Schaal, Sabine Salla, Jörg Timm, Gerd Geerling, Johannes Menzel-Severing

**Affiliations:** 1 Institute of Virology, Medical Faculty, Heinrich-Heine-University of Düsseldorf, 40225 Düsseldorf, Germany; lisa.mueller2@med.uni-duesseldorf.de (L.M.); philipp.ostermann@med.uni-duesseldorf.de (P.N.O.); schaal@uni-duesseldorf.de (H.S.); joerg.timm@med.uni-duesseldorf.de (J.T.); 2 Department of Ophthalmology, RWTH Aachen University, 52074 Aachen, Germany; ssalla@ukaachen.de; 3 Department of Ophthalmology, Medical Faculty, Heinrich-Heine-University of Düsseldorf, 40225 Düsseldorf, Germany; geerling@med.uni-duesseldorf.de

**Keywords:** SARS-CoV-2, COVID-19, corneal transplantation, eye banking

## Abstract

Concerns of possible transmission of SARS-CoV-2 from donors to patients by corneal transplantation have caused a decline in corneal transplantations. Graft culture media are routinely tested for infectious risks, but it is unclear whether this constitutes a viable means to avoid transmitting SARS-CoV-2 via keratoplasty. We found that SARS-CoV-2 RNA was not present in the medium after seven days of organ culture of corneas from donors (n = 4), who were SARS-CoV-2-positive upon tissue procurement. These medium samples showed no presence of viral RNA. To pursue this question under controlled conditions and further exclude the possibility of productive infection in corneal grafts, we inoculated corneoscleral discs from healthy donors (n = 8) with infectious SARS-CoV-2 and performed PCR testing of the culture medium at various time points. After seven days of culture, we also tested for SARS-CoV-2 RNA within the inoculated corneal tissue. The medium from tissue samples inoculated with SARS-CoV-2 showed no increase in viral RNA, which may indicate lack of viral replication in these corneal grafts. SARS-CoV-2-RNA was, however, detected on or in corneal tissue seven days after inoculation. Our data suggest that corneal grafts may not be permissive for replication of SARS-CoV-2 and demonstrates that PCR testing of culture media cannot safely exclude that tissue has been exposed to SARS-CoV-2. It also demonstrates the difficulty to differentiate between virus adherence and virus replication by PCR testing in SARS-CoV-2 exposed tissue.

## 1. Introduction

Since December 2019, the emergence of the novel beta coronavirus SARS-CoV-2 has been causing a devastating global pandemic that far surpasses its predecessors, SARS-CoV and MERS-CoV, in case numbers [1,2]. Although the novel coronavirus SARS-CoV-2, and its associated COVID-19 disease, are less lethal, its transmission rate is significantly higher compared to MERS-CoV or SARS-CoV [3,4]. With infections occurring worldwide, global research has shifted to quickly elucidating viral features and rapidly developing therapies and prophylactic vaccines. Understanding infection routes and identifying target tissues harboring a replication-competent virus have been high priorities since the beginning of the pandemic and continue to be of great importance, especially considering organ and tissue transplantation [5]. It has been established that SARS-CoV-2 enters target cells via interaction of its spike glycoprotein and the angiotensin-converting enzyme 2 (ACE2) receptor. Additionally, it requires proteolytic cleavage of the viral spike protein by host proteases, with type II transmembrane serine protease (TMPRSS2) being the main interaction partner [6]. Results from extensive RNA sequencing approaches and immunohistochemical analysis demonstrate the expression of both ACE2 receptor and TMPRSS2 on corneal and conjunctival epithelial cells [7,8], suggesting that the cornea might be targeted by SARS-CoV-2. Furthermore, the identification of Neuropilin-1 (Nrp-1) as an additional receptor that may be able to substitute for ACE-2, supports the idea of a broader tissue tropism of SARS-CoV-2 than previously assumed [9]. This raises the important question of whether transplantation of tissue from the ocular surface potentially puts corneal transplant recipients at risk of infection [10]. More so, because data from post-mortem swabs (conjunctival and nasopharyngeal) of patients deceased with COVID-19 suggest that these cannot reliably exclude infection in ocular tissue donors [11]. The detection of SARS-CoV-2 RNA [12] and protein [13] in ocular tissue post-mortem suggests that transmission of SARS-CoV-2 via a corneal transplant donor may be possible. Hence, a meaningful decline in corneal tissue provision was observed in different European countries during some phases of the pandemic [14,15]. A concomitant decline in the number of corneal transplantations was observed during a prolonged “lockdown” period in Italy [16]; this may at least in part be due to the cancellation of procedures considered non-urgent, although a reduction in tissue retrieval was also reported [17]. This is problematic, given a global shortage of donor tissue for sight-restoring corneal transplantation [18]. Therefore, it is important to elucidate SARS-CoV-2 tropism for corneal tissue. Available studies do not fully answer the question of whether ocular tissue is merely susceptible to SARS-CoV-2 or whether it is permissive, allowing for efficient virus replication. Additionally, it remains to be determined whether routine testing of media used for culturing quarantined donor corneas may be a viable means to identify infected tissue [19]. This study analyzed corneas from donors that died with COVID-19 and exposed corneal grafts to a replication-competent virus, while monitoring the supernatant for increasing viral RNA levels by qRT-PCR. 

## 2. Results

First, we investigated whether SARS-CoV-2 RNA could be detected in the culture supernatant of corneal grafts from deceased patients who were confirmed SARS-CoV-2 positive by PCR testing of nasopharyngeal swabs, obtained at the time of tissue procurement. After enucleation, cornea samples were stored in organ culture medium for seven days. At the end of the culture period, RNA was extracted from the supernatant and subjected to qRT-PCR to screen for SARS-CoV-2 RNA. Here, all samples (n = 4) showed no presence of viral RNA above the detection limit, suggesting no release of viral RNA into the medium and, hence, no presence of viral replication in these corneal grafts. 

**Figure 1:**
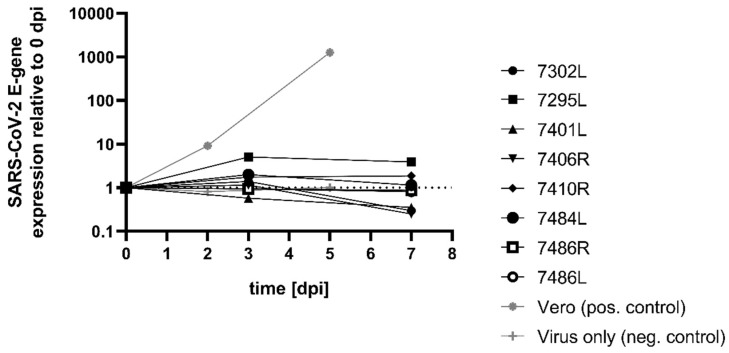
Tissue samples were inoculated with infectious SARS-CoV-2 at a TCID50/mL of 500–1000 (n = 8). Supernatant samples were taken at 0, 3, and 7 dpi or 0, 2, and 5 dpi for controls and subjected to SARS-CoV-2 E-gene specific qRT-PCR analysis to determine the amount of SARS-CoV-2 RNA expression (exp(−Δ*CT*) ratios normalized to 0 dpi).

To further test whether corneal grafts are permissive to SARS-CoV-2, we inoculated corneoscleral discs from healthy donors (n = 8) with an infectious SARS-CoV-2 B.1 isolate, at a tissue culture infectious dose 50 per mL (TCID50/mL), ranging between 500 and 1000. Medium samples were taken directly after inoculation to determine the amount of input RNA (0 days post-infection, dpi), as well as at 3 dpi and 7 dpi. As an approximation of viral replication, changes in the amount of viral RNA compared to 0 dpi were calculated (Figure 1). A general trend for all samples was that even after seven days, there was no increase in the amount of SARS-CoV-2 specific RNA, but it remained at a stable level comparable to that of the input sample at 0 dpi. As a positive control, SARS-CoV-2 permissive Vero cells were infected at a TCID50/mL of 500 and the amounts of viral RNA in the cell culture supernatant were measured at 0 dpi, 2 dpi, and 5 dpi. In contrast to the inoculation of cornea samples, qRT-PCR analysis media from the infected Vero cells showed a significant increase in viral RNA in the supernatant, which corresponds to a productive infection and viral replication. To test whether SARS-CoV-2 RNA remains stable in cell culture medium, a negative control (Virus only) was included where no cells were added. RNA levels did not differ at 2 dpi and 5 dpi compared to 0 dpi (Figure 1). 

**Figure 2:**
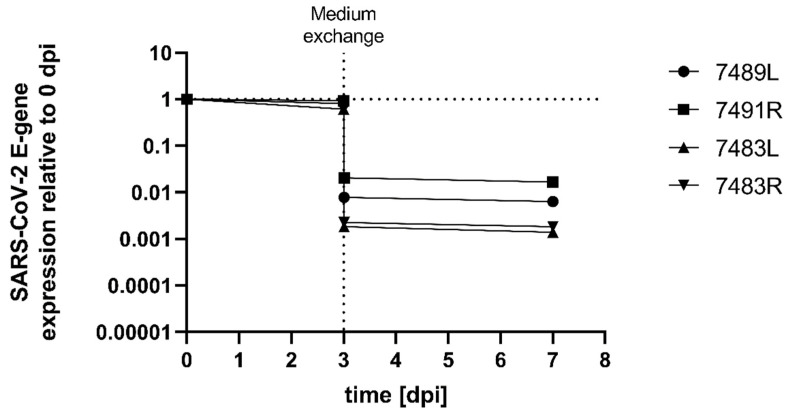
Tissue samples were inoculated with infectious SARS-CoV-2 at a TCID50/mL of 500–1000. Supernatant samples were taken at 0 and 3 dpi prior to medium exchange, 3 dpi post-medium exchange and at 7 dpi, and subjected to SARS-CoV-2 E-gene specific qRT-PCR analysis to determine the amount of SARS-CoV-2 RNA expression (exp(−Δ*CT*) ratios normalized to 0 dpi).

To further exclude low levels of SARS-CoV-2 replication in corneal grafts, we again inoculated tissue samples (n = 4) with virus-containing medium at a TCID50/mL ranging between 500 and 1000. Medium samples were taken: (i) directly after inoculation to determine the amount of input RNA (0 dpi); (ii) three days later prior to medium exchange (3 dpi w/o). Immediately after sample collection, the virus-containing medium was exchanged for fresh medium to minimize the overall background; and (iii) another sample was taken (3 dpi w). The culture period was stopped at (iv) 7 dpi and changes in the amount of viral RNA pre- and post-medium exchange were compared to 0 dpi (Figure 2). Again, no increase in the amount of viral RNA was detectable from 0 dpi to 3 dpi. As expected, after changing the virus-containing medium at 3 dpi, the amount of viral RNA was reduced, but was still detectable, suggesting that the detection of SARS-CoV-2 mRNA was mainly due to viruses that had not yet infected the ocular tissue. Thus, with further cultivation from (iii) to (iv) in fresh maintenance medium, no increase in the amount of viral RNA was observed. This suggests that no productive infection occurred during this experiment (Figure 2).

To evaluate the possibility that viral particles attached to or entered the corneal tissue but failed to replicate, we lysed the tissue of a subset of samples (n = 6) at 7 dpi after inoculation for elution via the EZ1 RNA Tissue Mini Kit, and subjected the eluates to qRT-PCR analysis to determine the amount of SARS-CoV-2 RNA within the tissue. Before lysis, all samples were washed twice in PBS to remove residual virus-containing medium. Interestingly, the amount of SARS-CoV-2 RNA in the tissue samples was comparable to that of the respective initial inoculation samples (Table 1), suggesting SARS-CoV-2 particles may be able to enter cells or attach to the tissue.

**Figure 3:**
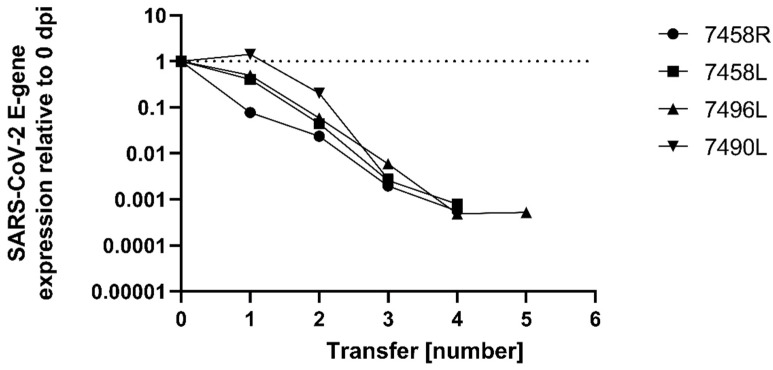
Tissue samples were inoculated with SARS-CoV-2 at a TCID50/mL of 500–1000. Every 24 h, tissue was transferred to fresh medium and supernatant samples were taken after each transfer and subjected to SARS-CoV-2 E-gene specific qRT-PCR analysis to determine the amount of SARS-CoV-2 RNA expression (exp(−Δ*CT*) ratios normalized to 0 dpi).

In the previous experiments, viral RNA was still detectable in the supernatant after medium exchange (Figure 2) and within the corneal tissue after washing steps (Table 1). Therefore, we included the following experiment: after an initial inoculation of the tissue samples (n = 4) with virus-containing medium for 24 h, tissue samples were transferred to fresh maintenance medium for further cultivation (Figure 3). In total, five transfer steps were carried out and the supernatant was analyzed for the presence and amount of viral RNA after each transfer. With each transfer, the amount of viral RNA decreased and was undetectable after the fourth transfer for most samples, showing that detection of viral RNA in the supernatant is likely caused by residual virus-containing medium that is transferred with the tissue rather than being caused by viral replication within the tissue (Figure 3).

## 3. Discussion

To allow for microbiological testing of the culture medium, organ cultured human donor corneas for transplantation are quarantined for one week (minimum five days) before being released for transplantation [20]. In this study, we took a homologous approach to testing the storage medium of donor corneas from patients who tested positive for SARS-CoV-2 at death. Our finding that no viral RNA was detected could be attributed to: (i) false negative results, (ii) the effectiveness of local disinfection with PVP Iodine [21], or (iii) the absence of productive infection of these corneas. Other authors did not detect SARS-CoV-2 RNA within the corneal tissue of 10 patients that died with COVID-19 [22], which supports the notion that corneal tissue is non-susceptible, despite the presence of entry receptors [8]. 

To test whether corneal transplants can be permissive to SARS-CoV-2 in a laboratory setting, we exposed the tissue to high levels of infectious virus that leads to productive infection in permissive cells and tissues [23,24]. Our observation that media samples showed no increase in viral RNA over an incubation period of one week (or no viral RNA at all after several media changes) is in line with results from recent studies on the effects of interferon on viral replication in corneal tissue, where replication of SARS-CoV-2 could not be detected in tissue samples [25]. These previous studies, however, were carried out using Optisol^TM^ medium and incubation was performed at 4 °C; this is the predominant corneal tissue culture system in North America, but it is rarely used in Europe [26]. Our results confirm and expand these findings to the organ culture system and testing of media. Along that line, we also analyzed levels of SARS-CoV-2 RNA in corneal tissue samples after the inoculation period with virus-containing medium. Tissue samples showed a high viral load at seven days post-inoculation despite the absence of productive infection, as determined by analyzing supernatant samples. Similarly, non-productive (abortive) SARS-CoV-2 infections have been described in neural tissue [24,27]. Here, the presented data only provide initial insights and require further dissection of viral attachment or entry mechanisms. Similar to the studies by Miner et al. [25], on the basis of our results we cannot identify the mechanism that is responsible for inhibiting a productive infection in corneal tissue.

Despite the finding that expression of ACE2 receptor is low in conjunctiva [28], active SARS-CoV-2 replication has been detected in conjunctival specimens [29]. Correspondingly, conjunctival secretions have been shown to contain SARS-CoV-2 RNA, especially in severely ill patients [30,31], and conjunctivitis related to COVID-19 can occur [32]. However, there is ongoing debate as to whether the conjunctiva can therefore act as a route of infection [33,34]. With regard to corneal tissue, this same question also warrants discussion since it will affect donation practice [35]. Although there are studies that report the full absence of SARS-CoV-2 RNA in corneal tissues obtained from a small number of COVID-19 positive post-mortem donors [36], other studies assessing the post-mortem presence of SARS-CoV-2 RNA in the corneal epithelium of 14 patients report that two patients with SARS-CoV-2 positive nasopharyngeal swabs also showed evidence of viral RNA in conjunctival swabs, while epithelium scrapes tested negative. These results further underline the importance of safety measurements in tissue procurement [37]. Viral infections that invariably prompt exclusion from cornea donation include hepatitis B and C, HIV, measles, rubella, rabies, Zika, and Ebola [20], despite the apparent absence of transmissibility of some of these viruses (e.g., HIV) through a corneal transplant [10]. Conversely, transmission of herpes simplex virus [38] and cytomegalovirus [39] have been reported, but specific measures to avoid the risk associated with these viruses are not widely adopted. This creates an ethical conflict between the Hippocratic principle “primum non nocere” and the unmet need for donor tissue in many regions [40]. With regard to SARS-CoV-2, our data add to the growing body of evidence suggesting that the risk of infection with SARS-CoV-2 in cornea recipients is low [14]. 

In conclusion, our data suggest that corneal grafts may not be permissive, that is, may not allow for SARS-CoV-2 to replicate, as determined by the qRT-PCR analysis of viral RNA in tissue culture supernatant. However, additional work is required to elucidate the factors that contribute to the suggested abortive infection and concomitant putative corneal resistance against SARS-CoV-2 replication in vivo and in vitro. We suggest that current recommendations for eye banks, such as adjusting eligibility criteria for corneal donation [37], be updated and revised as additional evidence emerges.

## 4. Materials and Methods

### 4.1. Cell Culture

Human donor corneoscleral discs unsuitable for transplantation were procured by the Aachen Cornea Bank (Department of Ophthalmology, RWTH Aachen University, Aachen, Germany) and by the Lions Eye Bank North Rhine-Westphalia (Department of Ophthalmology, University of Düsseldorf, Düsseldorf, Germany). Tissue was obtained within 72 h post-mortem after disinfection of the donation site with PVP iodine and stored at 34 °C in maintenance medium, composed of Eagle’s minimal essential medium (MEM) with 2% fetal bovine serum, penicillin, streptomycin, and amphotericin B, for a mean duration of 36 days (standard deviation: 12.3 days). 

### 4.2. Virus Inoculation 

Experiments involving infectious SARS-CoV-2 were performed within the biosafety level 3 laboratory at the University Hospital Düsseldorf. Infectious SARS-CoV-2 (B.1 isolate, EPI_ISL_425126) was added to the maintenance medium at a TCID50/mL of 500–1000 and samples were incubated at 37 °C, 5% CO_2_. TCID50/mL was determined on Vero cells (ATTC-CCL-81), as described in [24].

### 4.3. Supernatant Samples

To monitor viral replication, 100 µL of the cultures’ supernatant was taken at 0 dpi and subsequent timepoints. The supernatant was incubated with 400 µL AVL buffer (Cat No. 19073, Qiagen, Venlo, The Netherlands) for 10 min at room temperature and mixed with 400 µL 100% ethanol. RNA extraction from 200 µL of supernatant was performed using the EZ1 Virus Mini Kit v2. (Cat. no. 955134, Qiagen) following the manufacturer’s instructions to a final elution volume of 60 µL.

### 4.4. Tissue Samples 

After the incubation period, tissue samples were transferred to 6-well plates and washed twice with PBS before being subjected to RLT lysis buffer for elution, via the EZ1 RNA Tissue Mini Kit following the manufacturer’s instructions. Eluates were then processed as supernatant samples. 

### 4.5. qRT-PCR 

For further analysis, 5 μL of the eluate was subjected to qRT-PCR using the real-time TaqMan®-technique where a 113 base pair amplicon in the E-gene of SARS-Cov-2 was generated and detected according to [41], with minor modifications, as described by Ramani et al. (2020) [24]. We used the LightMix® Modular SARS and Wuhan CoV E-gene (Cat.-No. 53-0776-96) and the LightMix® Modular EAV RNA Extraction Control and the AgPath-ID® One-Step RT–PCR Kit (Applied Biosystems, Waltham, MA, USA, Cat. No. 4387391). PCR was performed on the ABI 7500 FAST sequence detector system (PE Applied Biosystems, Weiterstadt, Germany).

Gene expression analysis of GAPDH was performed to confirm the presence of a sufficient amount of cells in the samples as suggested in [42]. To this end, quantitative RT–PCR analysis was performed by using qPCR MasterMix (PrimerDesign Ltd, Chandler’s Ford, UK) with primers #5163 (5′ CCA CTC CTC CAC CTT TGA 3′) and #5164 (5′ ACC CTG TTG CTG TAG CCA 3′) and fluorescence emission was monitored by LightCycler 1.5 (Roche, Basel, Switzerland).

## Figures and Tables

**Table 1:** Tissue samples were washed and lysed before being subjected to qPCR analysis after inoculation with virus-containing medium. Shown here are the tissue ct-values in comparison to the ct-values of the initial inoculation. In addition, all samples showed a robust signal for GAPDH which was used as a housekeeping gene to confirm the presence of cells.

Sample	Tissue ct-Value	Supernatant ct-Value (Inoculation)
7489R	24,0	25,3
7491L	24,5	25,6
7401L	30,4	33,0
7484L	18,3	20,9
7486R	24,8	21,8
7486L	23,6	21,9

## Data Availability

The datasets generated during the study are made available from the corresponding author upon request.
